# Vagal control of the heart decreases during increasing imminence of interoceptive threat in patients with panic disorder and agoraphobia

**DOI:** 10.1038/s41598-021-86867-y

**Published:** 2021-04-12

**Authors:** Jan Richter, Anne Pietzner, Julian Koenig, Julian F. Thayer, Christiane A. Pané-Farré, Alexander L. Gerlach, Andrew T. Gloster, Hans-Ulrich Wittchen, Thomas Lang, Georg W. Alpers, Sylvia Helbig-Lang, Jürgen Deckert, Thomas Fydrich, Lydia Fehm, Andreas Ströhle, Tilo Kircher, Volker Arolt, Alfons O. Hamm

**Affiliations:** 1 Department of Biological and Clinical Psychology, University of Greifswald, Franz-Mehring-Str. 47, 17487 Greifswald, Germany; 2 Section for Experimental Child and Adolescent Psychiatry, Department of Child and Adolescent Psychiatry, Centre for Psychosocial Medicine, Heidelberg University, Heidelberg, Germany; 3 University Hospital of Child and Adolescent Psychiatry and Psychotherapy, University of Bern, Bern, Switzerland; 4 Department of Psychological Science, University of California, Irvine, Irvine, USA; 5 Department of Clinical Psychology and Psychotherapy, University of Marburg, Marburg, Germany; 6 Clinical Psychology and Psychotherapy, University of Cologne, Cologne, Germany; 7 Department of Psychology, Division of Clinical Psychology and Intervention Science, University of Basel, Basel, Switzerland; 8 Department of Psychiatry & Psychotherapy, Ludwig-Maximilians-Universität Munich, Munich, Germany; 9 Christoph-Dornier-Foundation for Clinical Psychology, Institute for Clinical Psychology Bremen, Bremen, Germany; 10 Department for Psychology & Methods, Jacobs University Bremen, Bremen, Germany; 11 Department of Psychology, School of Social Sciences, University of Mannheim, Mannheim, Germany; 12 Department of Psychology and Psychotherapy, University of Hamburg, Hamburg, Germany; 13 Department of Psychiatry, Psychosomatic Medicine and Psychotherapy, University of Würzburg, Würzburg, Germany; 14 Department of Psychology, Humboldt-Universität zu Berlin, Berlin, Germany; 15 Department of Psychiatry and Psychotherapy, Campus Charité Mitte, Charité-Universitätsmedizin Berlin, Berlin, Germany; 16 Department of Psychiatry and Psychotherapy, University of Marburg, Marburg, Germany; 17 Department of Psychiatry and Psychotherapy, University of Münster, Münster, Germany

**Keywords:** Emotion, Prefrontal cortex, Human behaviour, Diagnostic markers, Cardiovascular biology, Translational research

## Abstract

Theoretically, panic disorder and agoraphobia pathology can be conceptualized as a cascade of dynamically changing defensive responses to threat cues from inside the body. Guided by this trans-diagnostic model we tested the interaction between defensive activation and vagal control as a marker of prefrontal inhibition of subcortical defensive activation. We investigated ultra-short-term changes of vagally controlled high frequency heart rate variability (HRV) during a standardized threat challenge (entrapment) in n = 232 patients with panic disorder and agoraphobia, and its interaction with various indices of defensive activation. We found a strong inverse relationship between HRV and heart rate during threat, which was stronger at the beginning of exposure. Patients with a strong increase in heart rate showed a deactivation of prefrontal vagal control while patients showing less heart rate acceleration showed an increase in vagal control. Moreover, vagal control collapsed in case of imminent threat, i.e., when body symptoms increase and seem to get out of control. In these cases of defensive action patients either fled from the situation or experienced a panic attack. Active avoidance, panic attacks, and increased sympathetic arousal are associated with an inability to maintain vagal control over the heart suggesting that teaching such regulation strategies during exposure treatment might be helpful to keep prefrontal control, particularly during the transition zone from post-encounter to circa strike defense.

**Trial Registration Number**: ISRCTN80046034.

## Introduction

Panic attacks are defined as abrupt surges of intense fear that reach a peak within minutes during which four (or more) symptoms from a list of 13 symptoms occur (DSM-5)^1^. If such panic attacks are followed by persistent concerns about additional panic attacks or their consequences, a panic disorder is diagnosed. Most panic attacks are experienced during the day (85%) and outside the home (73%), thus, occurring in contexts that are typical for agoraphobic situations^2,3^ and, as a consequence, are often associated with agoraphobic avoidance.

Theoretically, panic attacks, anxious apprehension, and agoraphobic avoidance might be conceptualized as a dynamically organized cascade of defensive responses that vary systematically as a function of increasing proximity of threat^4–7^. When an organism enters a context in which a threat has been encountered previously but has not yet been detected, a class of adaptive behaviors is engaged, including increased vigilance, autonomic arousal and inhibition of appetitive behaviors. As soon as the threat is detected but is still distant, increased selective attentive freezing is evoked by the threatening cue. When the threat is coming closer, defensive actions such as fight or flight are mobilized^8–11^. In the case of panic disorder and agoraphobia, the survival-relevant threat, however, does not arise from external sources (e.g., a predator) but from inside the body. Potentially life threatening interoceptive challenges, such as cardiac arrhythmias or hypoxia, are among the primary symptoms of panic attacks^12,13^. Thus, defensive responses do not vary as a function of spatial distance and temporal proximity of exteroceptive threat cues. Instead the intensity and number of painful and non-painful interoceptive sensations often in combination with a perceived lack of control determine the defensive action evoked by interoceptive threat^6,14^. Consequently, acute panic attacks can be considered as instances of circa strike defense^5,6^ to intense interoceptive symptoms that seem to get out of control evoking feelings of extreme fear. These feelings are thought to be associated with a strong discharge of the sympathetic nervous system^15^, and the urge to escape^16^. Perception of mild bodily symptoms, often interpreted as precursors of an upcoming panic attack, evoke increased selective attention to such bodily symptoms and are associated with a potentiation of the startle reflex as an index of defensive freezing^17–19^ associated with post-encounter defense^6^.

The human brain comprises numerous networks to ensure the detection and discrimination of interoceptive signals that indicate danger for the body´s integrity and organize adaptive defensive responses^20–22^ including the internal regulation of visceromotor adaptations as described in the neurovisceral integration model (NIM) by Thayer and Lane^23,24^. According to this model, afferent information from all organs of the body are transmitted via afferent fibers of the vagus that terminate in the nucleus tractus solitaries (NTS). Visceral afferents from the heart, the blood vessels, the intestines, and the respiratory tracts that are relevant for detecting panic related symptoms terminate in the caudal part of the NTS. From there, information is projected via the central autonomic network (CAN)^25^ to the anterior cingulate, insular, and prefrontal cortex. Accordingly, increased activation in the anterior insula and cingulate cortex was found when individuals with high fear of body symptoms were exposed to cues previously associated with unpleasant body symptoms^18^. In addition to the afferent loop, the CAN is comprised of direct and indirect pathways that modulate the preganglionic sympathetic and parasympathetic neurons. As suggested in the NIM, the sympathoexcitatory, cardioacceleratory subcortical threat circuits that are activated during a panic attack are under tonic inhibitory control via GABA-ergic mediated projections from the prefrontal cortex^24,26^, which is hypothesized to be deactivated during threat processing. As elaborated in the NIM^24^ this tonic prefrontal inhibition can be indexed by the cardiac chronotropic control via a vagally mediated pathway and can be assessed by high frequency resting heart rate variability (HRV) with higher resting HRV being associated with stronger tonic prefrontal inhibition. Accordingly, phasic decreases of HRV reflect adaptive decreases of prefrontal control in favor of a more reflexive midbrain processing as observed in stress studies^27^.

Guided by this model, we analyzed the dynamics of phasic HRV changes during a potentially threatening context in patients diagnosed with a principal diagnosis of panic disorder with agoraphobia (PD/AG) and enrolled for a multicenter randomized controlled clinical trial^28,29^. Because marked fear of entrapment and avoidance of being in enclosed places is a prominent symptom in patients with PD/AG^30,31^ we analyzed vagally controlled HRV during and after a behavioral avoidance test (BAT; i.e., sitting in a small dark closed test chamber with the door locked from outside)^14^.

We hypothesized increasing deactivation of prefrontal inhibition with increasing imminent threat processing. Hence, we expected strongly decreased HRV during acute states of circa strike defense during the threat challenge as compared to a control condition. To test our hypothesis, we analyzed the phasic HRV changes during online-recorded panic attacks and prior to escape behavior during the BAT relative to a recovery phase after the BAT exposure. Together with decreased prefrontal inhibition and HRV we expected to observe relatively increased cholinergic sympathetic and cardioaccelatory activation during these instances of circa strike defense (i.e., immediately prior to escape and panic attacks) as measured by skin conductance level (SCL) and heart rates, respectively. Because the standardized exposure to a situation of entrapment provoked defensive activations in many but not all PD/AG patients^14^ we further hypothesized the BAT non-avoiding patients to show HRV changes depending on the intensity of distal threat processing. Following the aims of the Research Domain Criteria (RDoC) perspective^32^, we thereby used the phasic heart change during the exposure relative to the control condition to rank our non-avoiding patients into five groups (quintiles) of equal size along a distribution ordered by this autonomic reactivity measure of defensive mobilization. We expected that prefrontal inhibition would be deactivated as a function of threat proximity and, as a result, vagally mediated HRV would decrease with increasing heart rate. Because those non-avoiding patients managed to remain in this situation, we expected the HRV to increase during the BAT in high heart rate reactive patients (post-encounter defense) suggesting a recovery of prefrontal control. In this view it is also conceivable that a relative increase of prefrontal inhibition during BAT exposure would have suppressed defense mobilization from the start in low heart rate reactive patients (pre-encounter defense). Again, in an exploratory analysis, we also analyzed the relationship to other units of analysis, i.e., changes in SCL and reported fear to better understand the regulatory processes engaged during this standardized exposure exercise. We assumed associations between heart rate reactivity and both vagally controlled HRV and cholinergic sympathetic mediated SCL but considered the different units of analysis to be theoretically neutral in principle^32^ with a limited share of common variance due to the specific physiological basis.

## Method

### Participants

We tested defensive reactivity in a subgroup of *n* = 345 (259 female) PD/AG patients from a total sample of *n* = 369 patients^14^. 39 patients (11.3%) refused to enter the test chamber (passive avoiders) and, thus, could not be included in the analyses of the physiological data. 72 patients (20.9%) entered the test chamber but terminated the exposure prematurely (active avoiders). Of these, we could analyze physiological data from *n* = 46 patients (17 patients left the BAT during the very first minute of exposure; 4 patients had to be excluded due to cardio-active medications like β-blockers; for 5 patients no valid ECG data were available). Two hundred thirty-four patients remained in the test chamber for the entire exposure period. From these patients we could analyze physiological data from *n* = 186 patients (23 patients had to be excluded due to cardio-active medication; for 25 patients no valid ECG data were available). All included patients (*n* = 232 (171 female); mean age *m* = 33.9 years; *SD* = 10.5 years) were Caucasian and free from psychotropic medication.

Patient recruitment, inclusion and exclusion criteria of the overarching clinical trial are described elsewhere^28,29^ and briefly summarized in the Supplementary Methods. Patient enrollment occurred between July 2007 and January 2009. Patients gave written informed consent after receiving a detailed description of the study. The study was approved by the Ethics Committee of the Medical Faculty of the Technical University of Dresden, which was valid for all participating centers. The experimental procedures performed were in accordance with the relevant guidelines and regulations of the journal.

### Experimental stimuli, procedure, and data collection

Following inclusion of the patients, severity of psychopathology was evaluated during baseline assessment^29^. Patients participated in the BAT in each of the eight participating centers following baseline assessment and before treatment. The BAT procedure is described in detail elsewhere^14^. In short, the BAT consisted of a standardized exposure to a small (75 cm wide, 120 cm long, 190 cm high), dark and closed test chamber. The BAT consisted of three phases: anticipation (sitting in front of the opened test chamber for 10 min), exposure (sitting in the test chamber for an unknown and maximum duration of 10 min with the door locked from outside combined with the instruction to stay in the chamber for as long as possible but to knock on the door if there is the desire to leave the chamber), and recovery (sitting in front of the opened test chamber for 8 min). Intensity of experienced anxiety was assessed by paper and pencil after each phase on a 10-point [ranging from 1 (not at all) to 10 (very strong)] scale. Additionally, patients were instructed to press a button whenever they experienced a panic attack while being in the chamber and release the button as soon as the panic attack was over. Patients always had the opportunity to refuse to participate or to stop BAT exposure. Trained investigators immediately supported patients to handle the symptoms experienced if necessary.

A personal computer running VPM^33^ controlled stimulus presentation, button presses, and physiological data acquisition. Bioamplifiers recorded skin conductance level (SCL) and ECG as reported elsewhere^14^. In more detail, the ECG was recorded with two standard, electrolyte filled Ag/AgCl electrodes (8 mm diameter; Marquette Hellige) placed in an Einthoven Lead II configuration. Using a Coulbourn V75-04 bioamplifier (Allentown, PA) the raw signal was filtered online through an 8 to 13 Hz band-pass filter, amplified with the factor 5000 and continuously sampled at a rate of 1000 Hz and 100 Hz during the first five minutes of the recovery phase and the rest of the assessment, respectively. The output of the panic button press was digitized by an analog to digital (A/D) converter (Scientific Solutions, Mentor, Ohio) and recorded as a separate channel with the VPM software.

### Data reduction and analysis

After visual inspection of the ECG recordings to detect anomalous signals and movement artifacts, misplaced R-wave triggers were corrected whenever they had occurred using ANSLAB version 2.4 (Autonomic Nervous System Laboratory, University of Basel, Switzerland) to identify consecutive inter beat intervals (IBIs) in milliseconds. Heart rate was computed by converting the IBIs into beats per minute per half-second bins, which were averaged by blocks of 10 s. The IBIs were also used for a time domain analysis for ultra-short-term heart rate variability. Using an automated script in Stata 16 (StataCorp. 2019. College Station, TX: StataCorp LLC.) the root mean square of successive differences (RMSSDs) were calculated for blocks of 10 s each. This time window has already been demonstrated to be valid for the quantification of RMSSD^34^. IBIs corresponding to a mean heart rate below 40 bpm (IBI > 1500 ms) or above 200 bpm (IBI < 300 ms) were excluded from analyses. Further, any IBI greater than 10 times the inter quartile range from the median by subject were excluded from analyses. To adjust for deviations from a normal distribution and unequal variance, RMSSD was logarithmically transformed (natural log).

To control for possible confounding effects of high inter-individual differences within groups we range-corrected the SCL based on the individual maximum level. Due to missing values SCL data were not available in all patients (see figure legends for respective number of included patients). To evaluate the responses evoked by the BAT exposure we calculated the relative changes for each minute during the exposure phase relative to the mean scores of the recovery for all physiological variables.

To test our hypothesis of decreasing HRV with increasing defensive mobilization in non-avoiding patients, those patients who remained in the BAT (n = 186) were subdivided into five groups *based on their heart rate reactivity during BAT exposure* (heart rate during exposure minus heart rate during recovery) aiming for equivalent sample size (n = 37/38 each) within each reactivity group. As illustrated in Table 1 these five heart rate reactivity groups did not differ significantly in age, sex, smoking status, BMI, and severity of assessed pathology. To assess the influence of vagal control and sympathetic activation on heart rate reactivity during exposure we treated these groups as a between-subject variable and tested in a univariate analysis of variance for group differences in changes in RMSSD and SCL during exposure relative to recovery. In this first step, we decided to categorize the continuous variable in order to illustrate expected differences in the direction of RMSSD change (decrease vs. increase in high vs. low heart rate responders). Due to the possible limited validity of categorizing a continuous variable, the group analyses were complemented by correlation and regression analyses in the whole investigation sample including heart rate, RMSSD, and SCL.

**Table 1:** Distribution of gender, smoking status, age, BMI and symptom severity during baseline assessment in the six study groups.

	Heart rate reactivity groups in BAT non-avoiding patients					BAT active avoiding patients		
	1	2	3	4	5		Comparison between 5 non-avoiding groups	Comparison between group 5 and active avoiders
	(n = 37)	(n = 37)	(n = 38)	(n = 37)	(n = 37)	(n = 46)		
	n (%)							
Women	22 (59.5)	24 (64.9)	25 (65.8)	27 (73)	31 (83.8)	42 (91.3)	Chi^2^(4) = 6.14, *p* = 0.19	Chi^2^(1) = 1.09, *p* = 0.30
Smokers	18 (50)	13 (35.1)	14 (36.8)	20 (54.1)	12 (32.4)	14 (30.4)	Chi^2^(4) = 6.68, *p* = 0.23	Chi^2^(1) = 0.04, *p* = 0.85
	**Mean (SD)**							
Age (years)	32.59 (10.11)	35.59 (10.29)	34.92 (11.80)	35.46 (10.58)	33.19 (9.47)	31.76 (10.49)	*F*(4,181) = 0.64, *p* = 0.64	*F*(1,81) = 0.41, *p* = 0.52
BMI (kg/m^2^)	24.99 (5.14)	24.02 (3.87)	23.14 (3.21)	22.93 (3.60)	23.99 (3.86)	24.58 (5.69)	*F*(4,167) = 1.47, *p* = 0.21	*F*(1,74) = 0.26, *p* = 0.61
CGI (1–7)	5.43 (0.56)	5.30 (0.74)	5.26 (0.69)	5.35 (0.79)	5.27 (0.69)	5.22 (0.66)	*F*(4,181) = 0.38, *p* = 0.82	*F*(1,81) = 0.13, *p* = 0.72
SIGH-A (0–56)	24.30 (4.84)	24.41 (5.61)	24.21 (4.45)	24.65 (5.12)	23.81 (5.64)	25.07 (5.84)	*F*(4,181) = 0.13, *p* = 0.97	*F*(1,81) = 0.97, *p* = 0.33
Number of PA (0–4)	2.76 (2.35)	2.62 (2.61)	2.61 (2.22)	2.05 (2.11)	2.32 (2.27)	2.87 (2.42)	*F*(4,181) = 0.55, *p* = 0.70	*F*(1,81) = 1.09, *p* = 0.29
PAS (0–57)	29.02 (7.69)	28.75 (11.16)	26.37 (11.01)	27.11 (11.03)	26.23 (8.64)	28.95 (8.49)	*F*(4,181) = 0.64, *p* = 0.64	*F*(1,81) = 2.06, *p* = 0.16
MI Alone (1–5)	2.90 (0.75)	2.89 (0.76)	2.83 (0.71)	2.96 (0.98)	2.84 (0.78)	3.09 (0.88)	*F*(4,166) = 0.16, *p* = 0.96	*F*(1,72) = 1.64, *p* = 0.21
BDI-II (0–63)	18.08 (7.39)	15.89 (8.63)	15.03 (8.18)	17.24 (9.10)	17.27 (7.98)	16.76 (8.93)	*F*(4,181) = 0.82, *p* = 0.52	*F*(1,81) = 0.07, *p* = 0.79

Due to missing values, BMI scores, MI Alone scores and smoking status were available only for 213 patients (group 1: n = 33; group 2: n = 33; group 3: n = 36; group 4: n = 35; group 5: n = 35; active avoiders: n = 41), 211 patients (group 1: n = 36; group 2: n = 34; group 3: n = 34; group 4: n = 33; group 5: n = 34; active avoiders: n = 40) and 231 patients (group1: n = 36; group 2: n = 37; group 3: n = 38; group 4: n = 37; group 5: n = 37, active avoiders: N = 46), respectively. A patient was classified as a smoker if on average at least one unit of tobacco on each day during the last 4 weeks was reported. *BMI* Body Mass Index, *CGI* Clinical Global Impression Scale, *SIGH-A* Structured Interview Guide for the Hamilton Anxiety Scale, *Number of PA* number of panic attacks during the last week reported in the Panic and Agoraphobia Scale, *MI Alone* Mobility Inventory, alone subscale.

In a second step, we analyzed physiological changes in the group of active avoiders during the first minute of exposure and during the last minute prior to active escape from the test chamber to compare autonomic response patterns during more proximal and imminent threat and examine it relative to the high heart rate reactive but non-avoiding group. Finally, we tested autonomic response patterns during 18 online-recorded panic attacks for which physiological data were available during the 10 s block during which panic onset was marked by the panic button press and the respective 10 s blocks before and after panic onset. Again, dimensional analyses using correlation and regression analyses complemented the group comparisons.

Alpha level was set to 0.05. Greenhouse–Geisser corrections of degrees of freedom were applied whenever appropriate.

### Ethical approval

The RTC project was approved by the Ethics Committee of the Medical Faculty of the Technical University of Dresden (EK 164082006). The neuroimaging components were approved by the Ethics Committee of the Medical Faculty of the Rheinisch-Westfälische Hochschule University Aachen (EK 073/07). The experimental pharmacology study was approved by the Ethics Committee of the state of Berlin (EudraCT: 2006-00-4860-29).

## Results

### Non-avoiding patients

#### Heart rate

**Figure 1:**
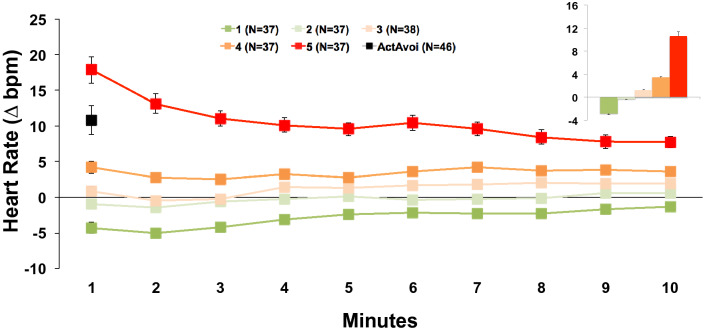
Heart Rate (means and standard errors) during ten minutes of acute threat exposure and averaged across the whole threat exposure (embedded figure) relative to a safe condition in n = 186 patients with no avoidance behavior during the task as a function of heart rate reactivity groups with increasing heart rate responses indicating increasing threat imminence. For comparison the responses during the first minute of exposure were also presented for n = 46 patients showing active avoidance but a minimum exposure duration of 60 s. Heart Rate in bpm, beats per minute.

As intended by group assignment the mean heart rate during the exposure linearly increased from group 1 to group 5 (F(4,181) = 164.19, p < 0.001, η_p_^2^ = 0.78; linear trend: p < 0.001; see Fig. 1). Group differences were most pronounced during the first minute and declined during the course of exposure (Time × Group F(36,1629) = 10.53, p < 0.001, η_p_^2^ = 0.19; linear trend Time × Group: p < 0.001) but were still significant during the last minute (F(4,181) = 45.87, p < 0.001, η_p_^2^ = 0.50).

#### RMSSD

**Figure 2:**
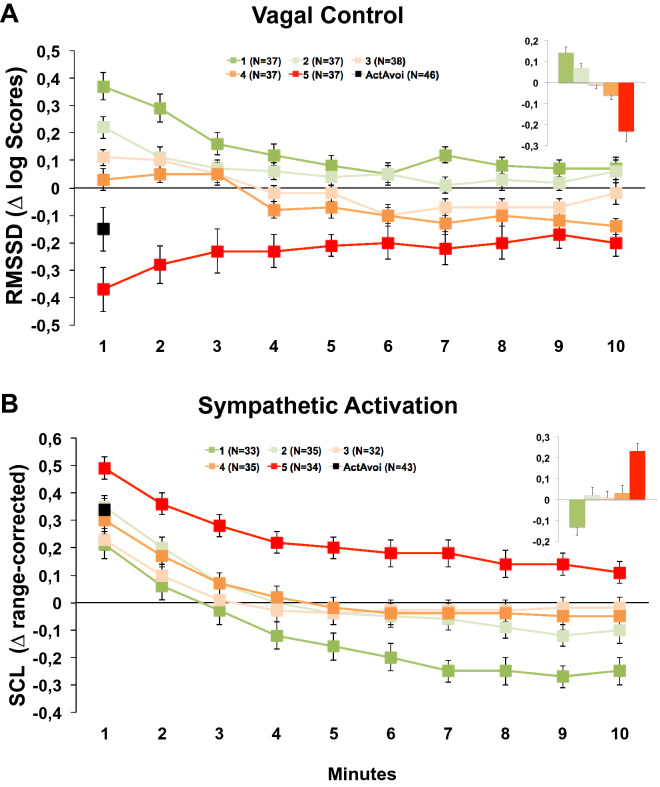
Vagal tone (**A**) and sympathetic activation (**B**; means and standard errors) during 10 mins of acute threat exposure and averaged across the whole threat exposure (embedded figure) relative to a safe condition in n = 186 patients with no avoidance behavior during the task as a function of heart rate reactivity groups with increasing heart rate responses indicating increasing threat imminence. For comparison the responses during the first minute of exposure were also presented for n = 46 patients showing active avoidance but a minimum exposure duration of 60 s. (**A**) Heart rate variability in logarithmic RMSSD scores, root mean square of successive differences. (**B**) Range corrected skin conductance levels (SCL) in 212 patients with available data.

The five groups also differed significantly in their RMSSD change scores (F(4,181) = 22.36, p < 0.001, η_p_^2^ = 0.22; linear trend: p < 0.001; see Fig. 2A). Patients showing the strongest increase in heart rate during exposure (group 5) exhibited the strongest reduction in RMSSD during exposure relative to the recovery period. In contrast, patients who showed no increase or even a reduction of heart rate during exposure relative to the recovery (group 1 and 2) showed a strong increase in vagal control during exposure which was most pronounced at the beginning of exposure. RMSSD differed most strongly between groups during the first minute of BAT exposure, decreased across exposure (Minute × Group F(36,1629) = 4.43, p < 0.001, η_p_^2^ = 0.09) but were still significant during the last minute (F(4,181) = 8.92, p < 0.001, η_p_^2^ = 0.17).

#### Skin conductance level

SCL also revealed significant group differences (F(4,164) = 11.58, p < 0.001, η_p_^2^ = 0.22), with elevated SCL relative to recovery in group five and reduced sympathetic arousal for group one when averaged across the entire exposure relative to recovery (see Fig. 2B). SCL significantly decreased during the course of BAT exposure in all five groups indicating a generally strong habituation within this response system (Time F(9,1512) = 154.71, p < 0.001, η_p_^2^ = 0.48). Nevertheless, group differences were still significant during the last minute of exposure (F(1,4) = 8.37, p < 0.001, η_p_^2^ = 0.17).

#### Reported fear

Fear ratings during the BAT relative to recovery also differed between groups (F(4,181) = 8.22, p < 0.001, η_p_^2^ = 0.15). The highest increase in reported fear was observed in group 5 followed by group 1 (group 1: m = 2.02, SD = 1.92; group 2: m = 1.17, SD = 1.22; group 3: m = 1.76, SD = 1.42; group 4: m = 1.89, SD = 1.94; group 5: m = 3.49, SD = 2.38).

#### Dimensional analyses

Dimensional analyses confirmed the results of the group comparisons. The overall heart rate change during BAT exposure relative to recovery was significantly negatively correlated with the overall change in RMSSD (r = − 0.60, p < 0.001), and positively correlated with increases in SCL (r = 0.51, p < 0.001). Importantly, changes of the heart rate response from the first minute of exposure to last minute of BAT exposure were significantly correlated with changes in the RMSSD across exposure duration (r = − 0.70, p < 0.001) but not with changes across time in the SCL, suggesting that changes in vagal control were more important for changes in heart rate than changes in sympathetic activation. Stepwise regression analyses confirmed these results with changes in RMSSD (beta = − 0.66, p < 0.001; overall ANOVA F(1,167) = 129.53, p < 0.001, R^2^ = 0.35) significantly predicting changes in heart rate across exposure while changes in SCL did not predict heart rate change during exposure.

### Imminent threat I: active avoidance

#### Between-group comparisons

We also analyzed the response during BAT exposure in those n = 46 patients showing active avoidance behavior after staying in the test room for at least one minute so that RMSSD could be reliably analyzed (mean duration of exposure: 282.37 s, SD = 158.14; range: 75–580 s). During the first minute of exposure, active avoiders showed a significant increase of their heart rate relative to recovery (m = 10.81, SD = 13.52; F(1,45) = 29.40, p < 0.001, η_p_^2^ = 0.40) which was, however, significantly lower as compared to the high reactive group 5 who completed the entire exposure session (see Fig. 1; F(1,81) = 6.28, p = 0.01, η_p_^2^ = 0.07). However, active avoiders showed high heterogeneity in heart rate changes (range: -5.92 to 41.53 ∆ bpm). The active avoiders also showed a significant reduction of the RMSSD during the first minute of exposure relative to the recovery phase (see Fig. 2A; F(1,45) = 3.90, p = 0.05, η_p_^2^ = 0.08) and an increase in SCL (see Fig. 2B; F(1,42) = 61.82, p < 0.001, η_p_^2^ = 0.60) but, again, changes were less pronounced as compared to completers group 5 (RMSSD: F(1,81) = 3.92, p = 0.05, η_p_^2^ = 0.05; SCL: F(1,75) = 6.18, p = 0.02, η_p_^2^ = 0.08). Again, heart rate changes were correlated with changes in RMSSD (r = − 0.79, p < 0.001) but not SCL. In contrast, the relative increase of reported fear was higher in BAT escapers as compared to completers group 5 (m = 4.69, SD = 2.10; F(1,81) = 5.96, p = 0.02, η_p_^2^ = 0.07).

#### Autonomic regulatory processes prior to escape

**Figure 3:**
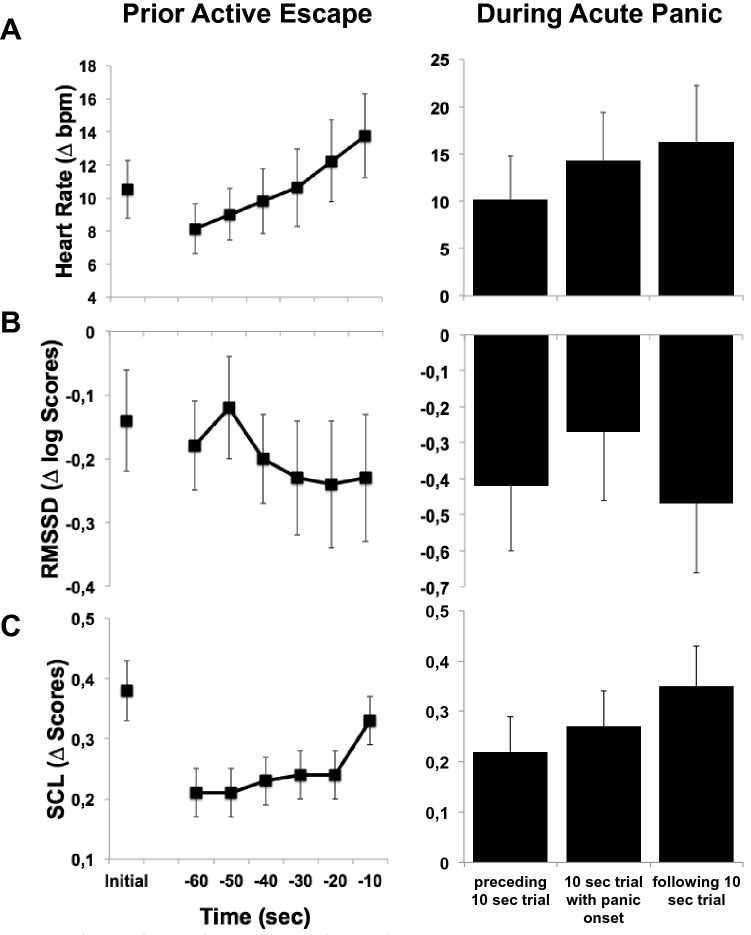
Physiological responses (means and standard errors) during imminent threat relative to a safe condition. Left column: The initial 10 s block of acute threat exposure and last six 10 s blocks prior to individual active avoidance behavior in n = 46 patients showing active avoidance behavior but a minimum exposure duration of 60 s. Right column: The 10 s block including individual panic attack onset and the respective block before and after in n = 18 recorded panic attacks. (**A**) Heart Rate in bpm, beats per minute. (**B**) Heart rate variability in logarithmic RMSSD scores, root mean square of successive differences. (**C**) Range corrected skin conductance levels (SCL). SCL data of 43 patients and 17 panic attacks were available prior active escape and during acute panic, respectively.

**Figure 4:**
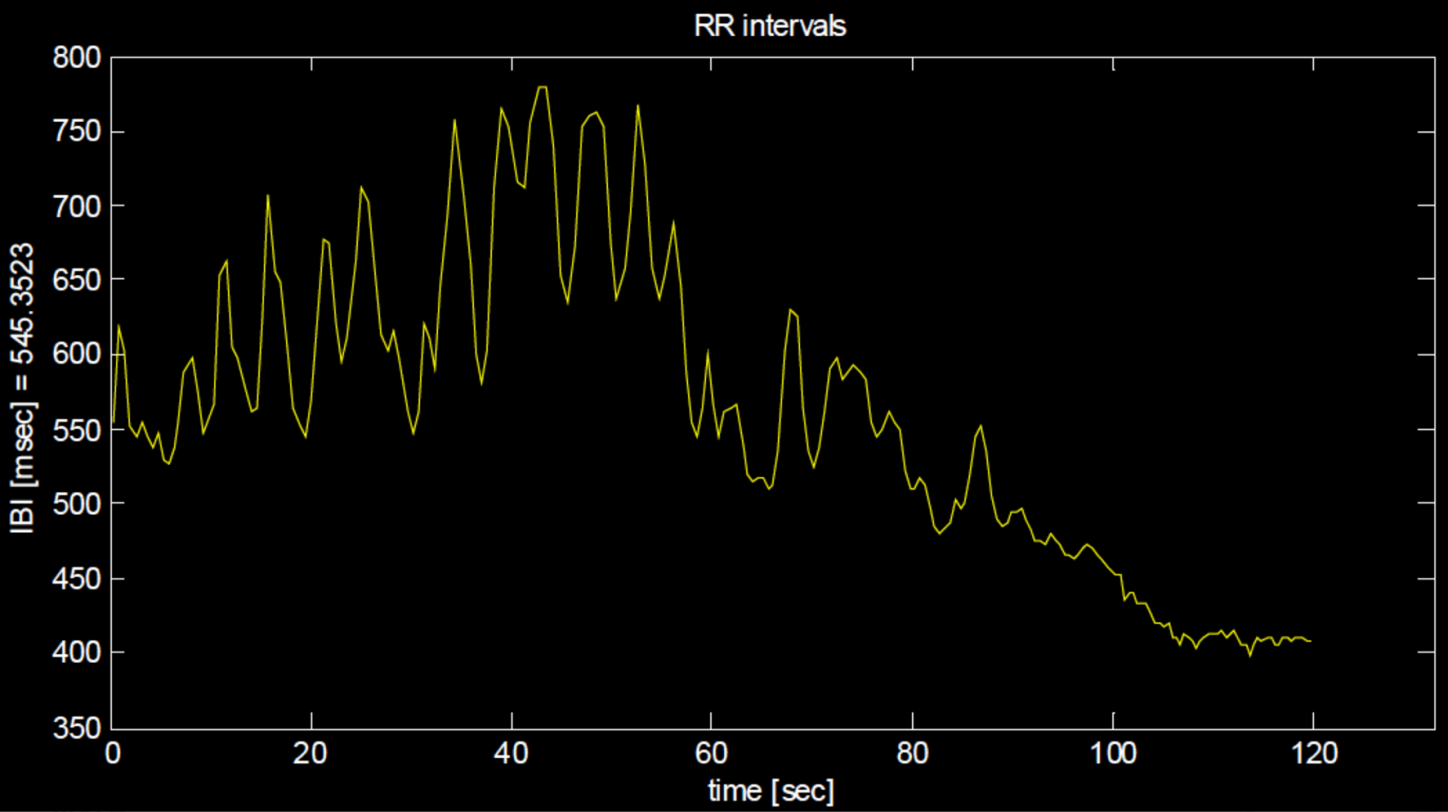
The inter-beat-intervals (IBIs) in ms as a function of time (s) in a single patient 120 s prior to escape from the chamber. Variability of IBIs, that is heart rate variability, first increased but then continuously decreased starting 60 s after onset of exposure and almost breaks off during the last 20 s prior to escape. The length of the IBIs also substantially decreased during this period to an average length of 410 ms, corresponding to a heart rate of 146 beats per minute, just prior to escape.

Figure 3 (left column) shows the mean scores and standard errors for changes in heart rate, RMSSD, and SCL, during the six last 10 s blocks just prior to escape from the test chamber and, for comparison, the first 10 s block at the beginning of BAT exposure. During the respective last minute of patients’ BAT exposure we observed a continuous increase of heart rate (F(5,225) = 6.98, p = 0.001, η_p_^2^ = 0.13; linear trend: p = 0.001), exceeding the heart rate increase at the beginning of exposure. The course of the RMSSD showed a different pattern with an initial increase in RMSSD during the first twenty seconds followed by a decrease in vagal control during the last 40 s prior to escape. This decrease in RMSSD just 40 s prior to escape was significantly negatively correlated with the increase in heart rate (r = − 0.48, p = 0.001). Differences in the heart rate responding during first minute of BAT exposure between active avoiders did not affect autonomic response patterns during last minute suggesting comparable regulatory processes immediately prior escape. However, lower initial heart rate reactivity and associated less pronounced reduction of RMSSD went along with higher overall increases of heart rate (r = − 0.45, r = 0.01) and decreases of RMSSD (r = 0.37, p = 0.01) from first to respective last minute of BAT exposure suggesting loss of vagal control before switching in circa strike defense although control was initially given. To illustrate this autonomic regulatory process on an individual level, Fig. 4 shows the course of the inter-beat intervals for one single patient 120 s prior to escape. As can be seen in this figure the patient increased vagal control over the heart at the beginning of the exposure session but has difficulties to maintain the vagal control after one minute before it completely breaks down 30 s prior to escape. As a consequence, inter-beat-intervals linearly decrease 60 s prior to escape. SCL did not change during the fifty seconds prior to escape but strongly increased during the last 10 s (F(1,42) = 25.19, p < 0.001, η_p_^2^ = 0.38; Fig. 3), suggesting that additional sympathetic activation is switched on just before the initiation of defensive action. This can also be seen in the individual case where vagal control is almost absent during the last 10 s prior to escape with no further increase in heart rate.

### Imminent threat II: acute panic

33 patients reported experiencing a panic attack during BAT exposure. Frequency of acute panic was minimal in non-avoiding groups 1 to 3 with almost no increase in heart rate (one patient per group), was low in group 4 with moderate increase in heart rate (n = 5, 13.5%) and moderate in group 5 (n = 9, 24.3%) with the strongest increase in heart rate. The highest frequency of acute panic was observed in active avoiders (n = 16, 34.8%; overall: χ^2^(5) = 31.28, p < 0.001).

We analyzed physiological response patterns in 18 online-recorded panic attacks (see Fig. 3, right column) during the 10 s block that included the button press (i.e., the reported onset of the panic attack) and the corresponding blocks before and after the reported onset. Heart rate started to increase prior to the button press and continued to rise during the panic attack (linear trend: F(1,17) = 7.20, p = 0.02, η_p_^2^ = 0.30). In contrast, RMSSD increased during the onset of the panic attack suggesting an effort for vagal regulation but collapsed during the panic attack as demonstrated by a subsequent decrease (quadratic trend: F(1,17) = 8.00, p = 0.01, η_p_^2^ = 0.32). In consequence, SCL linearly increased during acute panicking (linear trend: F(1,16) = 7.58, p = 0.01, η_p_^2^ = 0.32).

## Discussion

We investigated the interplay between prefrontal inhibitory vagal control and subcortical sympathoexcitatory regulation of the heart by re-analysing the physiological data of a group of PD/AG patients when exposed to a context in which the probability to experience interoceptive threat is elevated. We used a standardized exposure to a situation of entrapment which engages different stages of defensive activation in many—but not all patients with PD/AG^14^. That is why the analyses were based on data covering the entire dimension of threat responding along a continuum from safe to imminent threat without including a healthy control group who differ from the patient group in many other aspects than the lack of fear of entrapment. From the RDoC perspective our reanalysis is a study of the negative valence system as it relates to anticipated threat by physiological symptoms arising from inside the body. For these analyses we ranked the patients who remained in this situation for the entire period of time according to their heart rate response as an index of autonomic defensive reactivity relative to baseline into five groups (quintiles) of equal size. We used the average heart rate activity during recovery as the baseline because anticipation of the task is already associated with anxiety in some patients. In sum, our results confirmed our hypotheses that prefrontal control strongly decreased during clinical entities of circa strike defence that are acute panic attacks and active escape in PD/AG patients. Also, we found evidence that prefrontal inhibition initially decreased with increasing defensive mobilization in non-avoiding patients but partially recovered during BAT exposure. To the best of our knowledge, we have for the first time mapped the dynamics of prefrontal control in anxiety disordered patients during the in-vivo exposure to a potentially threatening and phobic context using phasic changes in vagally mediated HRV.

### Increase in vagal control to cope with uncertainty

Supporting our hypotheses, we found a strong inverse relationship between changes in heart rate during the exposure task and changes in HRV as an index of prefrontal vagal control^24,26^ both in the between group comparisons as well as in the dimensional correlation and regression analyses. This inverse association was most pronounced at the beginning of the exposure in all groups and declined throughout the ten-minute exposure duration. Those patients who showed strong heart acceleration at the beginning of exposure showed a substantial reduction of the RMSSD, suggesting that tonic inhibitory vagal control is deactivated when these patients enter a context in which potential interoceptive threat may occur. In contrast, those patients who showed even a reduction of their heart rate at the beginning of exposure exhibited a significant increase in their RMSSD. These data suggest that these patients actively increased their prefrontal inhibitory vagal control to decrease their heart rate during initial exposure. These group differences declined throughout the duration of exposure, suggesting that the effort to down-regulate cardiac acceleration by prefrontal control is reduced during the course of exposure, probably because patients felt safer in this context with no interoceptive threat occurring during the course of time. These findings support the idea put forward by Thayer and others^24,35^ suggesting that the default response to uncertainty, novelty and potential threat is sympathoexcitatory preparation that is inhibited through vagal control via prefrontal activation. According to the threat imminence or defense cascade model^5–7^ entering such a context in which a potential threat might be encountered this chronotropic control of the heart is also accompanied by general hypervigilance to all potentially dangerous cues that—in the case of PD/AG—arise from inside the body.

While changes in RMSSD significantly predicted changes in heart rate, changes in skin conductance did not, supporting the notion that vagal control is more prominent for regulating heart rate under these circumstances (i.e., pre-encounter defense). We also observed an increase in sympathetic activation particularly at the beginning of the exposure which, however, rapidly declined in all patients during the course of exposure, probably reflecting a rapid habituation process to the context. This pattern of autonomic regulation is different for those patients who prematurely terminated the exposure, i.e., showing maladaptive active avoidance behaviour.

### Collapse of vagal control and sympathetic activation during defensive action

Patients who prematurely terminated the exposure showed a linear increase in their heart rate starting already one minute before the flight response was activated. RMSSD scores were decreased relative to baseline supporting the view that the increase in heart rate was accompanied by reduced vagal control. RMSSD data also showed that patients tried to down-regulate their heart rate by enhancing prefrontal vagal control during the first ten seconds of the last minute prior active escape, which however, was not successful since heart rate continued to increase during this time period. As a result, vagal control decreased again resulting in a further increase in heart rate. Increase in sympathetic activation, as indexed by increases in skin conductance level did only occur during the last ten seconds just prior to initiation of the flight response. These data suggest that perception of body symptoms that seem to get out of control engage an autonomic and behavioural pattern that can best be described as a state of circa strike defense^5,6^. At the defensive transition zone to circa-strike threat^10^ vagal control collapses and increased sympathetic activation is switched on to support the effective flight response—in this case leaving the chamber as quickly as possible. During this preparation for escape there is also a strong inhibition of the startle response to acoustic probe stimuli thus shifting attention to the escape route and blocking the processing of irrelevant stimuli in the context of action^14,36,37^. Moreover, functional brain imaging data suggest that distributed activation shifts from prefrontal areas to midbrain when distal threats become more proximal^38–40^. The dynamic changes observed in the vagal control of the heart during increasing threat imminence would support these findings. In some patients vagal control seems to be completely blocked during preparation for escape. As compared to the high heart rate reactive non-avoiding patients group the reduction of RMSSD was less pronounced in active escapers during the first minute of BAT exposure but—in contrast to the non-avoiding patients—further increased, especially in those patients with initial high residual control demonstrating the strong dynamic of neurovisceral integration in the transition between distal and imminent threat processing.

The autonomic response pattern observed during preparation for escape was remarkably similar to the profile observed during spontaneous panic attacks during BAT exposure. Heart rate was accelerated ten seconds prior to the button press of reported panic attacks associated with a reduction of RMSSD relative to baseline. While heart rate further increased ten seconds after reporting a panic attack, RMSSD increased, suggesting that patients again tried to down-regulate their heart rate by increasing vagal control. While heart rate further increased during the next ten seconds up to an average of 14 beats per minute, RMSSD decreased again, indicating that vagal control of the heart was reduced. Sympathetic activation also increased linearly during the course of a panic attack as indicated by a linear increase in the skin conductance level. These data suggest that panic attacks can also be considered as an instance of circa strike defense, which are possibly triggered by brainstem circuits. This interpretation is supported by early reports^41^ showing that electrical stimulation of the PAG evoked acute panic attacks in humans^22^. Moreover, an increase in activation in the dlPAG during a respiratory challenge causing a feeling of dyspnea (inhaling CO_2_ enriched air) was found^42^. This increase in activation, however was stronger for panic disorder patients relative to healthy controls. Interestingly, this activation was reduced in a group of divers who have learned to actively cope with such respiratory challenges during their training, probably by recruiting prefrontal networks to downregulate the lower brainstem networks. The failure to get access to these networks might also increase the sense of losing control during panic attacks.

### Implications for therapy

The current findings might have implications for exposure-based therapies. A widely influential model describing the central mechanisms of change involved in exposure based therapies has been the emotional processing theory^13,43,44^. According to this theory three factors are important mechanisms of change, (a) initial fear activation, (b) fear reduction during and c) between exposure sessions. Therefore, it seems to be common sense that distraction procedures and safety signals during exposure exercise are often viewed as avoidance behaviour and are therefore considered to be counterproductive for these processes. The current data, however, suggest that teaching patients techniques to increase their vagal control over the heart might increase their capacity to keep prefrontal control over the brainstem systems, particularly during the critical transition zone from post-encounter to circa strike defense. In addition, using such regulation techniques might also induce some sense of control. Indeed, there is some evidence that breathing training might be helpful for treating patients with panic disorder^45^, however the application of such techniques during critical phases of exposure therapy have not been tested yet. First evidence that increased vagal control might be helpful to successfully complete exposure therapy comes from a study by Wendt and coworkers^46^ showing that higher cardiac vagal control prior to treatment reduced exposure treatment dropout in PDA patients. Thus, training of such “physiological regulation strategies” might increase prefrontal control and might increase inhibition of fear in such exposure contexts. First evidence from the laboratory that increased resting heart rate variability enhances inhibition of conditioned fear are promising^47,48^.

## Supplementary Information

Supplementary Information.

## Data Availability

All principle investigators take responsibility for the integrity of the respective study data and their components. All authors and co-authors had full access to all study data. Data analysis and manuscript preparation were completed by the authors and co-authors of this article, who take responsibility for its accuracy and content.
