# Supplementary material for: Vagal control of the heart decreases during increasing imminence of interoceptive threat in patients with panic disorder and agoraphobia
Source: Sci Rep. 2021 Apr 12;11:7960. doi: 10.1038/s41598-021-86867-y (PMC8041829; doi:10.1038/s41598-021-86867-y)
Supplement: Supplementary file 1 — Supplementary Information. [file 41598_2021_86867_MOESM1_ESM.docx]

**Vagal control of the heart decreases during increasing imminence of interoceptive threat in patients with panic disorder and agoraphobia**

Running Title: Cardiovagal control in panic disorder

Jan Richter^1^, Anne Pietzner^1^, Julian Koenig^2,3^, Julian F. Thayer^4^, Christiane A. Pané-Farré^1,^**^5^**, Alexander L. Gerlach^6^, Andrew T. Gloster^7^, Hans-Ulrich Wittchen^8^, Thomas Lang^9,10^, Georg W. Alpers^11^, Sylvia Helbig-Lang^12^, Jürgen Deckert^13^, Thomas Fydrich^14^, Lydia Fehm^14^, Andreas Ströhle^15^, Tilo Kircher^16^, Volker Arolt^17^, and Alfons O. Hamm^1^

**Supplementary Methods**

Patient recruitment, inclusion and exclusion criteria of the overarching clinical trial, as well as patients’ sociodemographic characteristics, comorbid diagnoses, and questionnaire data are described elsewhere (1,2). In short, eight German centers recruited patients from ongoing clinical mental health care, physician referral, and via additional advertisements in various media outlets. Inclusion criteria were a) a current DSM-IV-TR primary diagnosis of panic disorder and agoraphobia validated by standardized computer-administered personal Composite International Diagnostic Interview (CAPI-WHO-CIDI; DIAX-CIDI version; 3); b) a clinical interview score >=18 on the structured interview guide for the Hamilton anxiety scale (SIGH-A in anxiety and depression; 4); c) a score >=4 on the clinical global impressions scale (CGI, 5); d) age 18–65 years; e) ability and availability to regularly attend treatment sessions. Exclusion criteria were a) comorbid DSM-IV-TR psychotic or bipolar I disorder; b) current alcohol dependence/current abuse or dependence of benzodiazepine and other psychoactive substances; c) current suicidal intent; d) borderline personality disorder; e) concurrent ongoing psychotherapeutic or psychopharmacological treatment; f) physician-verified contraindications of exposure-based cognitive behavioral therapy.

**References**

1. Gloster AT, Wittchen H-U, Einsle F, Lang T, Helbig-Lang S, Fydrich T, et al. Psychological Treatment for Panic Disorder With Agoraphobia: A Randomized Controlled Trial to Examine the Role of Therapist-Guided Exposure In Situ in CBT. Journal of consulting and clinical psychology. 2011;79(3):406-20.

2. Gloster AT, Wittchen HU, Einsle F, Hofler M, Lang T, Helbig-Lang S, et al. Mechanism of action in CBT (MAC): methods of a multi-center randomized controlled trial in 369 patients with panic disorder and agoraphobia. European archives of psychiatry and clinical neuroscience. 2009; 259 Suppl 2: S155-66.

3. Wittchen H-U, Pfister H. DIA-X Interview. Instruktionsmanual zur Durchführung von DIA-X-Interviews (Instruction manual for the DIA-X-Interview). Swets & Zeitlinger, Frankfurt. 1997.

4. Shear MK, Vander Bilt J, Rucci P, Endicott J, Lydiard B, Otto MW et al. Reliability and validity of a structured interview guide for the Hamilton anxiety rating scale (SIGH-A). Depress and Anxiety. 2001; 13 (4): 166–178.

5. Guy W. Clinical global impression. In: Guy W (ed) ECDEU assessment manual for psychopharmacology, revised. National Insitute of Mental Health, Rockville, 1976; pp 217–222.
